# Site-specific resolution of enthesitis in patients with axial spondyloarthritis treated with tumor necrosis factor inhibitors

**DOI:** 10.1186/s13075-021-02534-7

**Published:** 2021-06-09

**Authors:** Michael J. Nissen, Burkhard Möller, Adrian Ciurea, Ruediger B. Mueller, Patrick Zueger, Martin Schulz, Fabiana Ganz, Almut Scherer, Eleftherios Papagiannoulis, Thomas Hügle

**Affiliations:** 1 Department of Rheumatology, Geneva University Hospital, 26 Avenue Beau-Séjour, 1211, Geneva 14, Switzerland; 2 Department of Rheumatology, University Hospital of Bern, Bern, Switzerland; 3 Department of Rheumatology, University Hospital Zurich, Zurich, Switzerland; 4 Division of Rheumatology and Immunology, Department of Internal Medicine, Kantonsspital St. Gallen, St. Gallen, Switzerland; 5 AbbVie Inc., North Chicago, IL USA; 6 AbbVie AG, Cham, Switzerland; 7 Swiss Clinical Quality Management in Rheumatic Diseases Foundation, Zurich, Switzerland; 8 Department of Rheumatology, Lausanne University Hospital (CHUV), Lausanne, Switzerland

**Keywords:** Axial spondyloarthritis, Enthesitis, Resolution, Tumor necrosis factor inhibitors

## Abstract

**Background:**

Enthesitis is a hallmark of spondyloarthritis (SpA) with a substantial impact on quality of life. Reports of treatment effectiveness across individual enthesitis sites in real-world patients with axial SpA (axSpA) are limited. We investigated the evolution of enthesitis following tumor necrosis factor inhibitor (TNFi) initiation in axSpA patients, both cumulatively and at specific axial and peripheral sites.

**Methods:**

AxSpA patients in the Swiss Clinical Quality Management Registry were included if they initiated a TNFi, had an available Maastricht Ankylosing Spondylitis Enthesitis Score, modified to include the plantar fascia (mMASES, 0–15), at start of treatment and after 6 and/or 12 months and ≥12 months follow-up. Logistic regression models were utilized to analyze explanatory variables for enthesitis resolution.

**Results:**

Overall, 1668 TNFi treatment courses (TCs) were included, of which 1117 (67%) had active enthesitis at baseline. Reduction in mMASES at the 6- and 12-month timepoints was experienced in 72% and 70% of TCs, respectively. Enthesitis resolution at 6/12 months occurred in 37.9%/43.0% of all TNFi TCs and 40.7%/50.9% of first TNFi TCs. At 6 months, a significant reduction in the frequency of enthesitis was observed at all sites, except for the Achilles tendon and plantar fascia among first TNFi TCs, while at 12 months, reduction was significant at all sites in both TC groups. Enthesitis resolved in 60.3–77% across anatomical sites, while new incident enthesitis occurred in 4.0–13.5% of all TNFi TCs at 12 months. Both baseline and new-incident enthesitis occurred most frequently at the posterior superior iliac spine and the fifth lumbar spinous process. Younger age and lower mMASES at baseline were predictors of complete enthesitis resolution, while female sex and second- or later-line TNFi treatment were associated with persistence of enthesitis at 12 months.

**Conclusion:**

In real-world axSpA patients treated with a TNFi, enthesitis improved in the majority of patients across all anatomical sites. Significant improvement at the Achilles and plantar fascia entheses was observed only at 12 months. Complete and site-specific enthesitis resolution occurred in ≥40% and ≥60% of TCs evaluated at 12 months, with a low incidence of new site-specific enthesitis.

**Trial registration:**

Not applicable.

## Key points

1. Enthesitis is present in approximately two-thirds of axial SpA patients in a real-world cohort at the time of initiation of a TNF inhibitor
2. After 12 months of therapy with a first TNFi, more than 50% of patients experienced complete resolution of enthesitis.
3. While enthesitis improved across all anatomical sites, resolution occurred more slowly at the Achilles tendon and the plantar fascia.

## Introduction

Axial spondyloarthritis (axSpA) is an inflammatory rheumatic disease with a diverse clinical presentation [1, 2]. AxSpA is characterized by excess bone formation that results in bone fusion and sclerosis of the sacroiliac joints and spine [1–4]. Chronic inflammatory back pain is the most common symptom of axSpA, and the associated pain, stiffness, and fatigue limit physical functioning and the ability to perform activities of daily living [2, 5]. Other musculoskeletal manifestations of axSpA include arthritis, dactylitis, and enthesitis [1, 2]. Extra-articular manifestations, such as acute anterior uveitis, psoriasis, and inflammatory bowel disease are also characteristic of axSpA [1, 2]. Consequently, axSpA carries a significant patient burden and has a substantial impact on patients’ quality of life [1, 2, 5–8].

Enthesitis, defined as an inflammation of the tendon, ligament, and/or joint capsule insertions into bone, is reported in the majority of patients with spondyloarthritis (SpA) [9] and is recognized as the distinctive pathological process in SpA [10, 11]. Advanced imaging and studies in animal models and humans have shown that enthesitis is associated with diffuse effects on adjacent connective tissue and underlying bone structures [11, 12]. Pathophysiological mechanisms of enthesitis in SpA may include both mechanical and autoimmune features [13, 14]. Repeated biomechanical stress is thought to cause microdamage at the entheses, which in turn induces an inflammatory response in the adjacent synovial tissue leading to synovitis [11, 13, 14]. Enthesitis is associated with higher disease activity, more disability, work absenteeism, and a poorer quality of life in axSpA patients [9].

Conventional synthetic disease-modifying anti-rheumatic drugs (csDMARDs) have been used with some success to treat peripheral arthritis in SpA patients; however, they have not clearly demonstrated efficacy for the treatment of enthesitis or the axial manifestations of axSpA [15–17]. In contrast, tumor necrosis factor inhibitors (TNFi’s) have been shown to be effective in improving or resolving enthesitis in clinical trials in patients with axSpA [18–23]. However, other clinical trials evaluating the benefit of TNFi’s on enthesitis in axSpA did not demonstrate a significant improvement or reported results that varied by the assessment utilized [24, 25]. It is important to note that many of these studies tended to involve a rather small number of patients, with a low baseline prevalence of enthesitis and with enthesitis as a secondary outcome. Real-world data on the effectiveness of TNFi’s for enthesitis in axSpA patients is even more limited. Moreover, data on enthesitis site-specific treatment effectiveness is rare, but required because the effectiveness of TNFi’s on enthesitis may vary by enthesitis site and therefore vary depending on the enthesitis index utilized [25]. The objective of this analysis was to investigate the real-world treatment effectiveness of TNFi therapy on enthesitis, both cumulatively and at specific enthesitis locations, including the spine, thoracic cage, Achilles tendon, and the plantar fascia, in patients with axSpA initiating a TNFi.

## Methods

### Study design and data source

This observational cohort study utilized prospectively collected data from the nationwide Swiss Clinical Quality Management in Rheumatic Diseases (SCQM) registry in inflammatory rheumatic diseases. This ongoing cohort of patients with ankylosing spondylitis/axSpA was established in 2005 [26] and provides an integrated feedback system for rheumatologists and their patients to monitor disease activity, disability, and radiographic damage using standardized assessments [27, 28]. At registry inclusion, demographic and disease characteristics, concomitant treatments, laboratory values, and comorbidities were collected by the treating rheumatologists [26]. Patients completed self-administered questionnaires to assess their disease state and quality of life [26]. Informed written consent was obtained from all patients prior to inclusion in the SCQM registry, and a regional ethics committee (CER-VD, 2019-00278) provided approval for collection of patient data from the SCQM cohort [26].

### Participants

Included patients were ≥16 years of age with a diagnosis of axSpA, as determined by a consultant rheumatologist, and had initiated a TNFi after inclusion into the SCQM registry. Patients were required to have available enthesitis assessments (i.e., modified MASES [mMASES]; modified to include the plantar fascia) at TNFi initiation (“baseline visit”; visits with a mMASES within 90 days prior to TNFi treatment initiation start date were considered valid), ≥1 enthesitis assessment available during the follow-up period, and a treatment course (TC) with ≥1 year of follow-up after treatment initiation. It was therefore possible for a single patient to contribute several TCs to the analysis. TCs with a particular TNFi (e.g., with a change of dose or a brief pause in treatment) in the same patient were merged if they corresponded to the same TNFi and if the end of the last dose of the last TC was within a 90-day time window of the first dose of the next TC. The follow-up period for a TC was defined as the time interval between treatment initiation day until the latest of the following days: last recorded visit, last recorded treatment stop date, or last recorded treatment adjustment date.

### Outcomes

The mMASES included the 13 sites of the MASES: 1st costochondral joints, 7th costochondral joints, iliac crests, anterior superior iliac spines, posterior superior iliac spines, insertion of Achilles tendons, and the 5th lumbar spinous process, as well as 2 additional sites, with inclusion of the plantar fascia insertion bilaterally, for a total of 15 sites (range, 0–15) [29]. Among patients presenting with enthesitis at baseline (mMASES≥1), both enthesitis resolution (mMASES=0) and mMASES score reduction at 6- and 12-month follow-ups were assessed both overall and in the subset of TCs who were TNFi naïve at baseline (i.e., first TNFi TC). Enthesitis localization frequencies at the 15 mMASES sites were examined at 6-month and at 12-month follow-ups.

### Statistical analyses

Baseline demographic and clinical characteristics were analyzed using descriptive statistics; continuous variables were summarized as medians with IQR, and categorical variables as frequencies and percentages of patients. Regarding mapping of patient information to baseline, 6-month and 12-month follow-up timepoints, we considered visits within the following intervals: baseline (0 to −90 days), 6 months (±90 days), and 12 months (±90 days) respectively. For enthesitis localization change, an intention to treat (ITT) approach was used whereby TCs were analyzed regardless of whether the patient was still under the same treatment at 6 and/or 12 months. Additionally, we performed a sensitivity analysis following a per protocol (PP) approach whereby we only considered the subset of TCs where the patient remained on the initial treatment at 12 months. McNemar’s test or a Mid-*p* method (if counts in the contingency tables were <25) was used to compare the proportion of patients with enthesitis at each mMASES site between baseline and 6 or 12 months, and the resultant *P* values were corrected for multiplicity using the Holm-Bonferroni method (correction was performed per set of comparisons for the 2 follow-up timepoints and *P*<0.05 level of significance was utilized).

Multiple missing value imputation by chained equations (MICE) was performed for baseline covariates for all TNFi TCs. The missingness for these variables varied from 1–15% for disease duration, height, weight, HLA-B27, and ASAS classification, to 20–30% for exercise score and ASDAS-CRP. In addition to the most important variables presented in Table 1, mMASES at follow-up timepoint was also included in the imputation model. No evidence was found that any of the baseline variables influenced the missingness distribution and a missing-at-random (MAR) data pattern was assumed. The MICE algorithm was run with 75 imputations and 30 iterations. Diagnostic measures were used to evaluate the convergence of the MICE algorithm and distribution of imputed values. We utilized logistic regression in order to analyze the binary outcome of enthesitis resolution and odds ratios were derived. The analyses were performed with both MICE imputed data and complete case data.

**Table 1:** Baseline characteristics of treatment courses for axSpA patients initiating a TNFi

Variable	All TC (*n*=1668 TCs)	All TC with mMASES ≥1 at baseline (*n*=1117)	All TC with mMASES = 0 at baseline (*n*=551)
Age (years), median (IQR)	42 (33–51)	43 (34–51)	41 (32-51)
Men, n (%)	886 (53.1)	531 (47.5)	355 (64.4)
BMI, median (IQR)	25.3 (22.5–28.6) *n*=1408	25.7 (22.8–29.3) *n*=932	24.7 (22.3-27.3) *n*=476
Disease duration (years), median (IQR)	9 (4–18) *n*=1633	9 (4–17) *n*=1092	10 (4-19) *n*=541
HLA-B27+, n (%)	984 (65.7) *n*=1498	617 (61.5) *n*=1003	367 (74.1) *n*=495
ASAS axial SpA criteria positive, n (%)^a^	1158 (76.4) *n*=1515	764 (76.2) *n*=1003	396 (77.2) *n*=513
Enthesitis (mMASES >0) at baseline, n (%)	1117 (67.0)	1117 (100)	-
mMASES, median (IQR)	2 (0–4)	3 (2–6)	-
Elevated CRP, n (%)	987 (60.1) *n*=1642	641 (58.4) *n*=1098	346 (63.6) *n*=544
ASDAS-CRP^b^, median (IQR)	3.3 (2.6–3.9) *n*=1220	3.4 (2.8–4.0) *n*=816	3.0 (2.3-3.7) *n*=404
BASDAI score^c^, median (IQR)	5.7 (4.1–7.0) *n*=1337	6.1 (4.7–7.3) *n*=889	4.6 (2.9-6.2) *n*=448
Ever experienced uveitis, n (%)	258 (16.5) *n*=1567	146 (13.9) *n*=1054	112 (21.8) *n*=513
Ever experienced arthritis, n (%)	963 (57.7)	707 (63.3)	256 (46.5)
Ever experienced dactylitis, n (%)	214 (12.9) *n*=1655	168 (15.0) *n*=1108	46 (10.3) *n*=447
Ever experienced enthesitis at the heel, n (%)	745 (69.5) *n*=1072	625 (73.0) *n*=856	120 (55.6) *n*=216
csDMARD cotherapy, n (%)	392 (23.5)	274 (24.5)	118 (21.4)
TNFi line of therapy, n (%)			
First line	1046 (62.7)	702 (62.9)	346 (62.8)
Second line	389 (23.3)	261 (23.4)	128 (23.2)
≥Third line	233 (14.0)	154 (13.8)	77 (14.0)

^a^Patients without ASAS axial SpA criteria positive were either ASAS axial SpA criteria negative or unknown due to missing variables

^b^ASDAS disease activity states: inactive (<1.3), low (≥1.3 to <2.1), high (≥2.1 to ≤3.5), very high (>3.5)

^c^BASDAI scores range from 0 (no disease activity) to 10 (maximum disease activity)

*ASAS* Assessment of SpondyloArthritis international Society, *ASDAS* Ankylosing Spondylitis Disease Activity Score, *BASDAI* Bath Ankylosing Spondylitis Disease Activity Index, *CRP* C-reactive protein, *csDMARD* conventional synthetic disease-modifying antirheumatic drug, *HLA-B27* human leukocyte antigen B27, *IQR* interquartile range, *mMASES* modified Maastricht Ankylosing Spondylitis Enthesitis Score (modified to include the plantar fascia), *TCs* treatment courses, *TNFi* tumor necrosis factor inhibitor

## Results

**Fig. 1:**
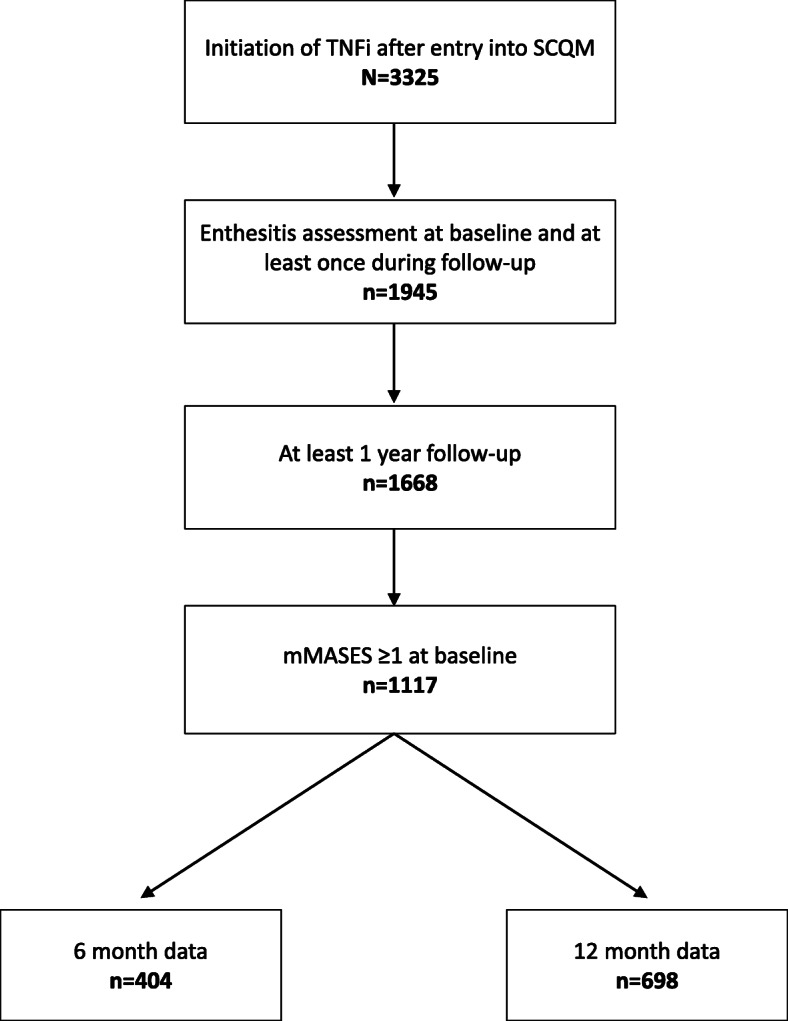
Selection of axSpA Study Sample. Number of treatment courses for each category are shown. MASES, Maastricht ankylosing spondylitis enthesitis score; SCQM, Swiss Clinical Quality Management; TNFi, tumor necrosis factor inhibitors

Of 3325 identified TCs in 2130 patients with axSpA who initiated TNFi therapy, 1668 TCs in 1393 patients met all 3 entry criteria of a mMASES available at baseline, at least another mMASES reported during follow-up, and available follow-up of at least 12 months (Fig. 1). The mean number (SD) of visits from baseline (0 to −90 days) to 12 months (± 90 days) in the group with non-missing mMASES data was 2.7 (1.8). Regarding the TCs, at baseline, the majority included men (53.1%), median age was 42 years (IQR, 33–51), median disease duration was 9 years (IQR, 4–18), and median Ankylosing Spondylitis Disease Activity Score (ASDAS-CRP) was 3.3 (IQR, 2.6–3.9) (Table 1). The median mMASES was 2 (IQR, 0–4) and 67% of TCs (1117 of 1668) were enthesitis positive (mMASES ≥1) at baseline; 23.5% were simultaneously co-treated with a csDMARD, and most TCs included patients receiving first-line TNFi therapy (62.7%). At baseline, TNFi’s initiated included adalimumab (30.2%), golimumab (23.7%), etanercept (23.6%), infliximab (18.0%), and certolizumab (4.4%). Baseline characteristics (Table 1) demonstrated that in the subgroup with enthesitis at baseline there was a lower proportion of men, lower rates of HLA-B27 positivity, and higher BASDAI values, whereas age, BMI, disease duration, and ASAS axial SpA criteria positivity were similar between those with and without baseline enthesitis.

**Fig. 2:**
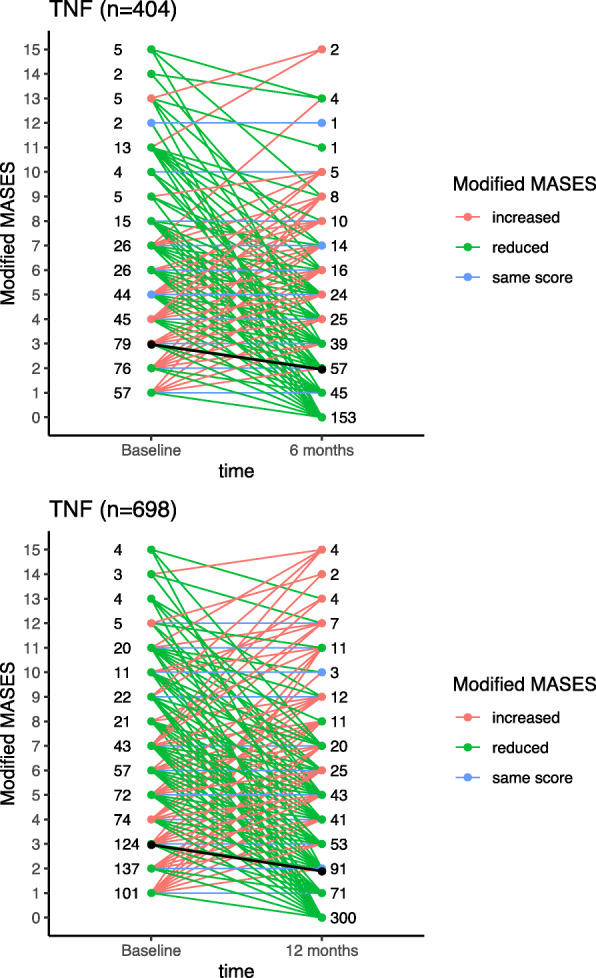
mMASES at Baseline and (A) 6 or (B) 12 Months. Each line on the plot connects the before and after mMASES values of a treatment course. More than one treatment course may have overlapping lines. The line colours indicate whether mMASES reduced, increased, or stayed the same. The numbers at baseline and the 6-month and 12-month time points indicate the number of TCs with the corresponding score. The black line indicates the evolution of the mean mMASES score between the 2 time points. For the assessment of modified MASES at the 6-month time point, values in interval [6 ± 3 months] were used and at the 12-month time point, values in interval [12 ± 3 months] were used. mMASES, modified Maastricht ankylosing spondylitis enthesitis score (modified to include the plantar fascia); TC, treatment course; TNFi, tumor necrosis factor inhibitor

Regarding TNFi TCs for patients with active enthesitis at baseline (*n*=1117) and available enthesitis assessments at 6 months (*n*=404) or 12 months (*n*=698), a reduction in mMASES was experienced by 72% and 70%, respectively. Score distributions at baseline and at the 6- and 12-month timepoints are presented in Fig. 2A and B. Similar findings were observed in the first TNFi TC subgroup (data not shown). Mean mMASES (SD) decreased from 2.9 (3.2) at baseline to 1.9 (2.8) at 6 months and to 1.8 (2.8) at 12 months in the all TC group and from 2.8 (3.0) at baseline to 1.6 (2.5) at 6 months and to 1.5 (2.7) at 12 months in the first TNFi subgroup.

Complete resolution of enthesitis (mMASES=0) was observed in 153/404 patients (37.9%) at 6 months and 300/698 patients (43.0%) at 12 months in the group including all TCs, compared with 98/241 (40.7%) and 234/460 (50.9%), respectively, in the first TNFi subgroup. Estimates from the PP analyses were almost identical in direction, effect size, and significance to the ITT approach.

**Fig. 3:**
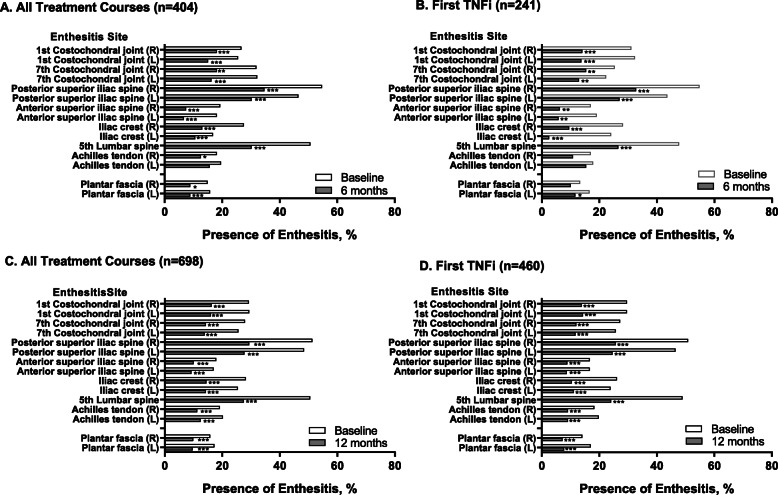
Enthesitis Localization at Baseline and 6 (A, C) or 12 (B, D) Months. Patients had active enthesitis at baseline and received TNFi therapy. Data are presented for all TCs and for the subgroup of first TNFi TCs. P value corrected for multiplicity (using the Holm-Bonferroni method). TC, treatment course; TNFi, tumor necrosis factor inhibitor. *P<0.05, **P<0.01, and ***P<0.001

**Fig. 4:**
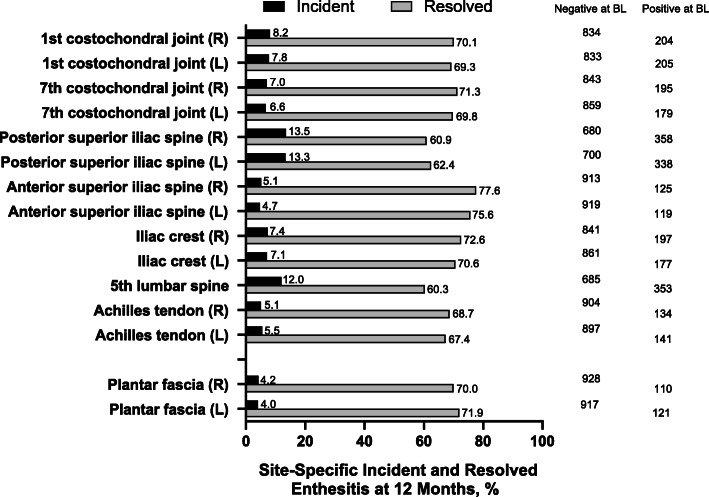
Incident and Resolved Enthesitis by Location at 12 months. Enthesitis by location was assessed for all TNFi treatment courses with non-missing follow-up data at 12 months (n=1038). BL, baseline; TNFi, tumor necrosis factor inhibitors

At baseline, enthesitis was most frequently observed at the fifth lumbar spinous process and the posterior superior iliac spine and least frequently at the plantar fascia (Fig. 3). In patients with active enthesitis at baseline, there was a significant reduction from baseline to 6-month follow-up in the frequency of enthesitis at all observed sites, except for the left Achilles tendon when including all TNFi TCs (Fig. 3A), and across all sites, except for the right and left Achilles tendons and the right plantar fascia in the first TNFi subgroup (Fig. 3B). At 12-month follow-up, there was a significant reduction from baseline in the frequency of enthesitis at all observed sites in both the all TC group (Fig. 3C) and the first TNFi subgroup (Fig. 3D). Across enthesitis sites evaluated, enthesitis resolved in 60.3–77%, while new incident enthesitis occurred in 4.0–13.5% of all TNFi TCs at 12 months (Fig. 4). Incident enthesitis, like baseline enthesitis, most frequently occurred at the posterior superior iliac spine and the fifth lumbar spinous process, whereas incident enthesitis least frequently occurred at the plantar fascia, the anterior iliac crest, and the Achilles tendon.

**Fig. 5:**
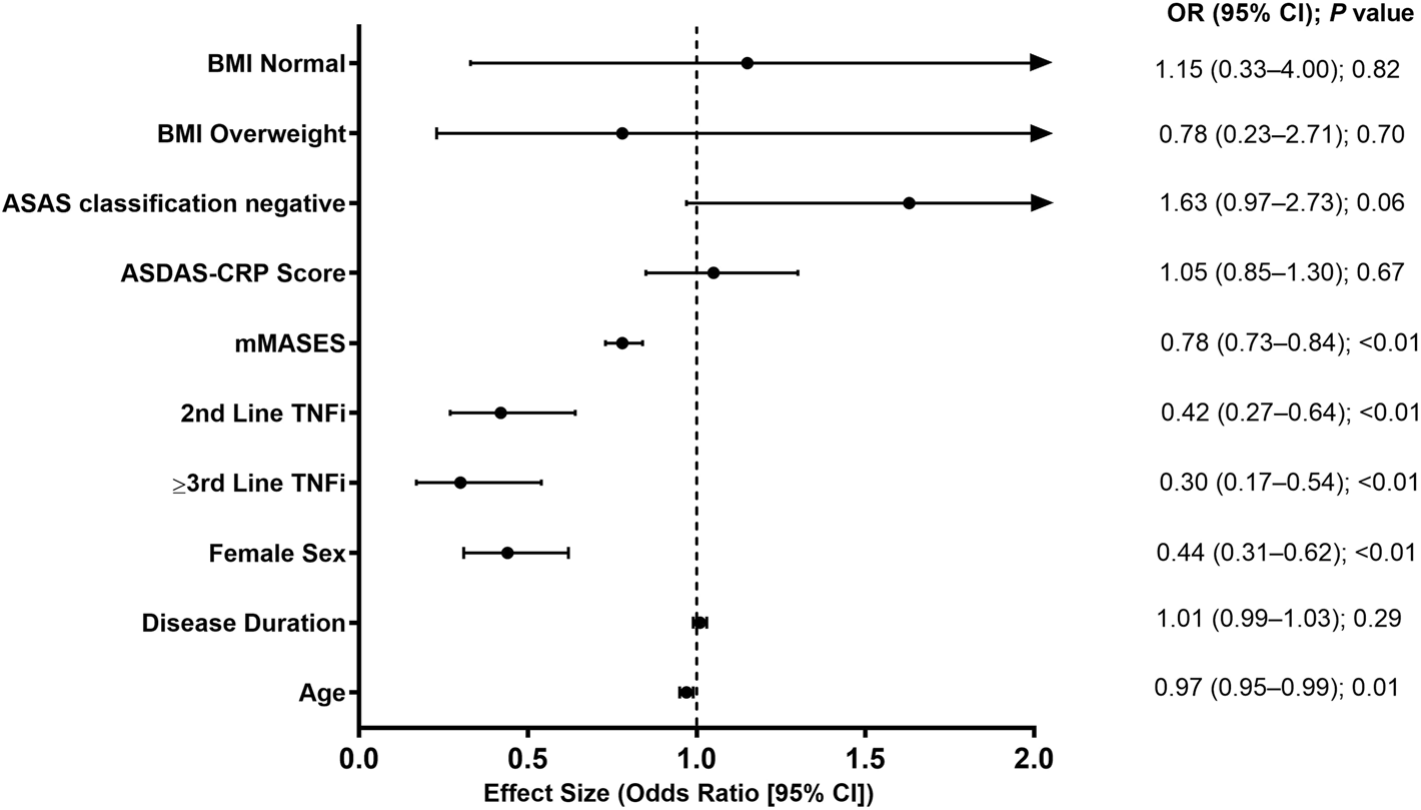
Explanatory Variables of Enthesitis Resolution at 12-Month Follow-up (n=698). Logistic regression analysis for resolution of enthesitis based on multiple imputation of missing baseline covariate data. Analysis was performed with 698 treatment courses in patients who initiated TNFi and with available enthesitis assessments at 12-month follow-up. The numbers of enthesitis resolutions observed at 12-month follow-up was 300. All variables presented represent values at baseline. BMI is a categorical variable with levels defined as underweight (BMI <18.5), normal (BMI 18.5–24.9), and overweight (BMI >24.9). ASAS classification negative indicates patients not meeting ASAS criteria for axial spondyloarthritis. Predictors with 95% CIs that extend beyond the x-axis scale upper limit of 2 are indicated with an arrowhead at the end of the error bar. ASDAS-CRP, Ankylosing Spondylitis Disease Activity Score–C-reactive protein; ASAS, Assessment of SpondyloArthritis international Society; BMI, body mass index; mMASES, modified Maastricht ankylosing spondylitis enthesitisscore (modified to include the plantar fascia); TNFi, tumor necrosis factor inhibitor

At the 12-month follow-up, younger age and lower mMASES at baseline were predictors of complete enthesitis resolution, while female sex and second- or later-line TNFi treatment were associated with persistence of enthesitis at 12 months in the MICE pooled estimates (Fig. 5). Negative Assessment of SpondyloArthritis international Society (ASAS) classification criteria status at baseline showed a trend favoring enthesitis resolution in the MICE analysis (*P*=0.06), which was not observed in the complete case analysis (P=0.2). The MICE pooled 6-month estimates were similar to the 12-month estimates, with younger age, lower mMASES, and negative ASAS classification criteria status at baseline being associated with the resolution of enthesitis, while female sex was associated with the persistence of enthesitis.

## Discussion

Although many clinical trials have supported the use of TNFi’s for the treatment of enthesitis [22, 23, 30], the effectiveness of TNFi’s for enthesitis in patients with axSpA in a real-world setting is largely unknown. In this analysis of real-world axSpA patients using data from the SCQM registry, we demonstrate that the mMASES decreased in 72% and 70% of patients at the 6- and 12-month timepoints, respectively. Complete resolution of enthesitis (mMASES=0) occurred in 37.9% and 43.0% of all TCs at 6 and 12 months, respectively, and in 40.7% and 50.9% of first TNFi TCs at 6 and 12 months, respectively. A possible explanation for the difference in enthesitis resolution between 6 and 12 months could be that longer TNFi treatment leads to a better response. Overall, in this axSpA patient population, a significant decrease in the frequency of enthesitis involvement was observed at all mMASES sites after 12 months, suggesting the real-world effectiveness of TNFi for the treatment of enthesitis.

It is interesting to note the differences in the improvement of site-specific enthesitis from baseline to 6 months and baseline to 12 months, whereby a significant improvement from baseline to 6 months was observed at all sites, except the Achilles tendon and the plantar fascia, which was particularly evident for the subgroup with a first TNFi. In contrast, all entheseal sites, including the Achilles tendon and the plantar fascia, demonstrated significant improvement from baseline to 12 months. This suggests that other factors, such as weight-bearing activities and higher mechanical stress at the entheses around the ankle, may lead to the requirement for a longer period of TNF inhibition in order to see a significant clinical improvement.

Data on incident enthesitis in SpA patients is very limited and largely restricted to patients with psoriatic arthritis. In patients with peripheral SpA included in the ABILITY-2 trial, 3.6% of patients treated with adalimumab presented new-onset enthesitis of the Achilles tendon at 12 weeks [18], which is similar to the rate of 5.3% in our population of axial SpA. Conversely, a significantly higher proportion of patients (10.9%) in the placebo group presented new-onset enthesitis at the Achilles tendon in ABILITY-2 [18]. New-onset enthesitis at the insertion of the plantar fascia was observed in 4.8% of treated patients in the ABILITY-2 trial (and in 8.7% of the placebo group) [18], which once again is very similar to the rate of 4.1% in our population. The higher rates of incident enthesitis observed at the 5th lumbar spine and the posterior superior iliac spine may in part be a reflection of non-inflammatory processes, such as degenerative disease of the lumbar spine.

The mean mMASES in our study population of 2.9 (SD, 3.2) was somewhat lower than that generally observed in randomized trials, such as the RAPID-axSpA trial with mean MASES scores of 4.7 in radiographic-axSpA patients and 5.6 in non-radiographic-axSpA patients [22]. Nevertheless, in our cohort, there were significant reductions of mean mMASES over 12 months to 1.8 in the all TC group and to 1.5 in the first TNFi subgroup. The RAPID-axSpA trial reported resolution rates of enthesitis of 53.8–55.4% in radiographic-axSpA patients and of 47.1–49.3% in nonradiographic-axSpA patients at 24 weeks [22], which are similar to the rates of 38–51% in our study. The RHAPSODY trial [23] reported resolution of plantar fascia enthesitis in 70.5% of patients at week 12, which is similar to the findings for our population at 12 months (resolution in 70.0–71.9% of TCs).

Patients with many positive enthesitis sites may be more likely to present with concomitant fibromyalgia, and the clinical overlap of these conditions may be more frequent in women than in men with axSpA [31, 32]. AxSpA patients with concomitant fibromyalgia could be expected to respond less efficiently to TNFi. In the overall population of the current study, 47/1668 patients presented with a diagnosis of concurrent fibromyalgia, including 8 men (1.0%) and 39 women (5.0%). The rate of fibromyalgia in the subgroup with a mMASES≥1 at baseline was 3.5%. These proportions of fibromyalgia are somewhat lower than estimates in the literature that range from 4 to 25% in axSpA patients [33].

This current study has a number of strengths, most importantly that it is the largest real-world study to date evaluating the effectiveness of TNFi on enthesitis in axSpA patients. While there are some data on the evolution of enthesitis in routine clinical practice following treatment with TNFi’s in both psoriatic arthritis (PsA) and peripheral SpA, there are almost no real-world data regarding axial SpA. The fact that we utilized the MASES is also advantageous, as in axial SpA, this score has demonstrated better correlation with BASDAI and BASFI than the Leeds Enthesitis Index [34]. In addition, we include data from both 6- and 12-month timepoints, which is important as enthesitis may resolve more slowly than other manifestations of axial SpA. In our manuscript, we report information on both overall incident enthesitis, as well as incident enthesitis by site, which is rarely described.

One of the limitations of this analysis is that no direct control group was available, and therefore, we cannot make any definitive statements about the causal effect of TNFi treatment on enthesitis reduction or resolution. Registry data are limited to the information collected at each visit, and patients may not have follow-up visits at timepoints of interest, making it more challenging to assess the effectiveness of treatments. It is unlikely that the ASAS axial SpA classification criteria-negative patients in our cohort in fact presented PsA, as in Switzerland there is a separate cohort for PsA and, additionally, cutaneous psoriasis was present in only 4% of the study population.

We hereby demonstrate that in a large real-world population of patients with axial SpA treated with a TNFi, a reduction in enthesitis was experienced by at least 70% of patients, while the complete resolution of enthesitis at 12 months was experienced by more than half of patients receiving a first TNFi. Significant reduction of enthesitis was observed at all sites at 6 months, except for the Achilles tendon and the plantar fascia, and at all sites at 12 months, suggesting that the ankle entheses may be more prone to mechanical strain and therefore require additional time for resolution. Finally, we describe the predictors of complete enthesitis resolution, which could be useful knowledge for the clinician initiating a TNFi.

## Importance and relevance of the study

We describe the real-world effectiveness of TNFi therapy on enthesitis in patients with axial SpA, both cumulatively and at specific enthesitis locations. The variability of the therapeutic response based on the localization of the enthesis and the factors predictive of resolution should be useful knowledge for the clinician initiating a TNFi.

## Data Availability

The datasets used and/or analyzed during the current study are available from the corresponding author on reasonable request.

## Abbreviations

ASAS: Assessment of SpondyloArthritis international Society
ASDAS-CRP: Ankylosing Spondylitis Disease Activity Score
axSpA: Axial spondyloarthritis
csDMARDs: Conventional synthetic disease-modifying antirheumatic drugs
IQR: Interquartile range
ITT: Intention to treat
MASES: Maastricht Ankylosing Spondylitis Enthesitis Score
mMASES: Modified Maastricht Ankylosing Spondylitis Enthesitis Score
MICE: Multiple missing value imputation by chained equations
SCQM: Swiss Clinical Quality Management in Rheumatic Diseases
SpA: Spondyloarthritis
TNFi: Tumor necrosis factor inhibitor
